# Supplementary material for: Iron limitation in M. tuberculosis has broad impact on central carbon metabolism
Source: Commun Biol. 2022 Jul 9;5:685. doi: 10.1038/s42003-022-03650-z (PMC9271047; doi:10.1038/s42003-022-03650-z)
Supplement: Supplementary file 2 — Supplementary Material [file 42003_2022_3650_MOESM2_ESM.pdf]

Iron limitation in *M. tuberculosis* has broad impact on central carbon metabolism.

SUPPLEMENTAL FIGURES

BIORENDER PUBLICATION RIGHTS

Monique E. Theriault[1], Davide Pisu[1], Kaley M. Wilburn[1], Gabrielle Lê-Bury[1], Case W. MacNamara[2], H. Michael Petrassi[2], Melissa Love[2], Jeremy M. Rock[3], Brian C. VanderVen[1], and David G. Russell[1*].

[1]Department of Microbiology and Immunology, College of Veterinary Medicine, Cornell University, Ithaca, NY, USA

[2]California Institute for Biomedical Research (Calibr), La Jolla, CA, USA

[3]Department of Host-Pathogen Biology, The Rockefeller University, New York, NY, USA

*Corresponding Author:

David G. Russell

Microbiology and Immunology

College of Veterinary Medicine

Cornell University, Ithaca, NY 14853

Tel 607 253 4272

Email: dgr8@cornell.edu

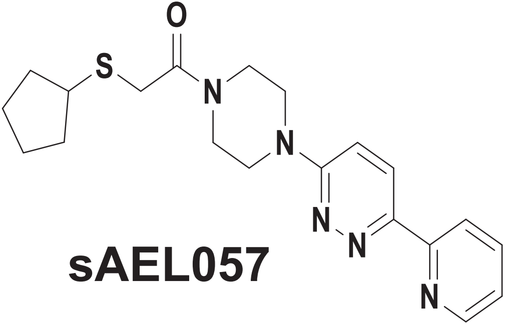

sAEL057

Supplemental Figure 1. Structure of sAEL057

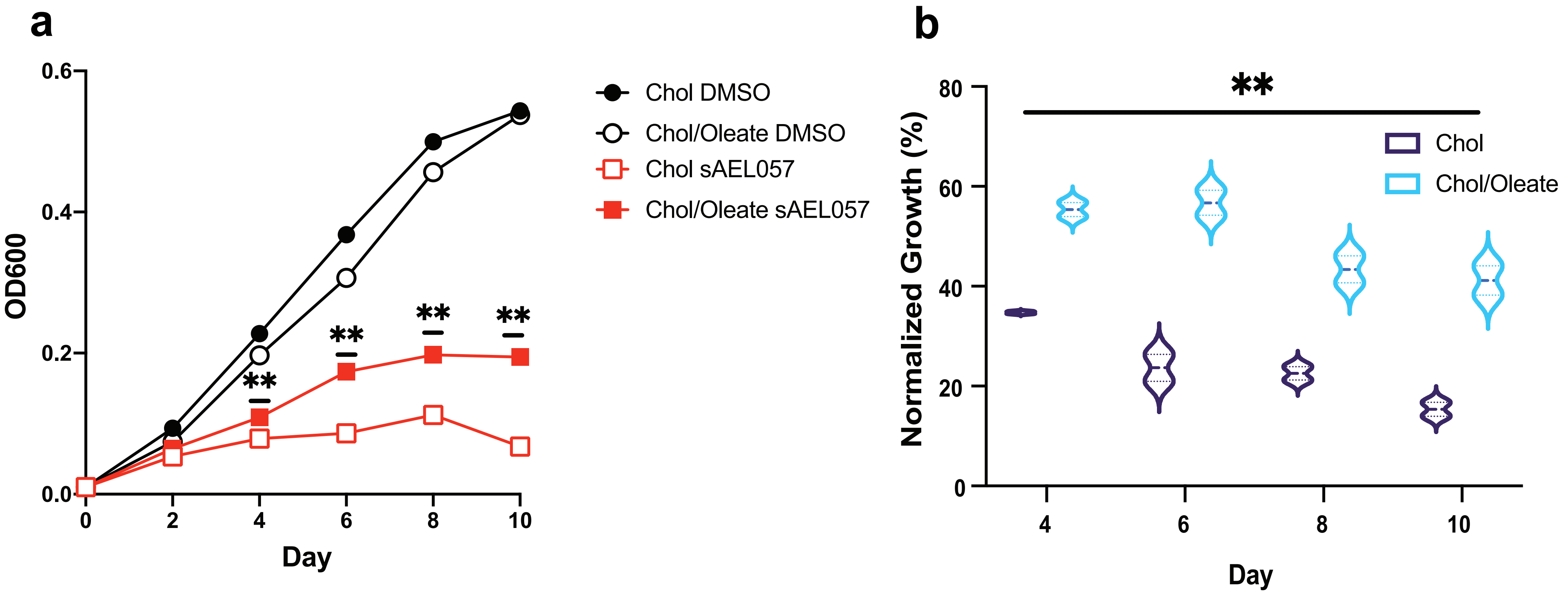

Supplemental Figure 2. Oleate partially rescues growth defect of sAEL057 treatment in cholesterol media. **a** Growth curves of Mtb grown in either cholesterol (chol) or cholesterol + oleate (chol/oleate) and treated with 10 μM sAEL057 or DMSO control. **b** Normalized growth comparing sAEL057 treated bacteria in media containing cholesterol or cholesterol + oleate. Statistical significance was assessed using a student's unpaired t-test.

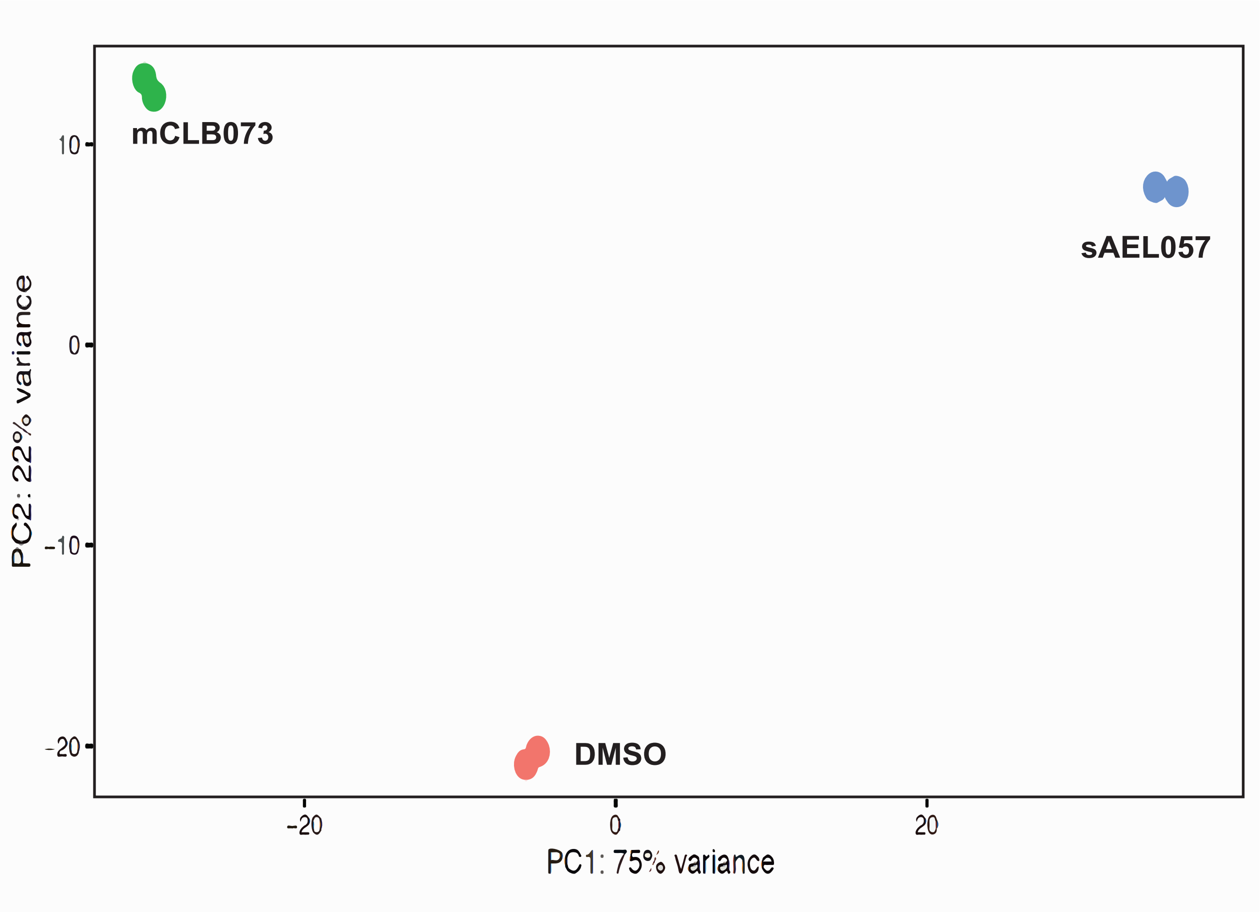

Supplemental Figure 3. sAEL057 treatment causes large shift in Mtb gene expression. Principal component analysis (PCA) of Mtb transcriptomes from growth in cholesterol media.

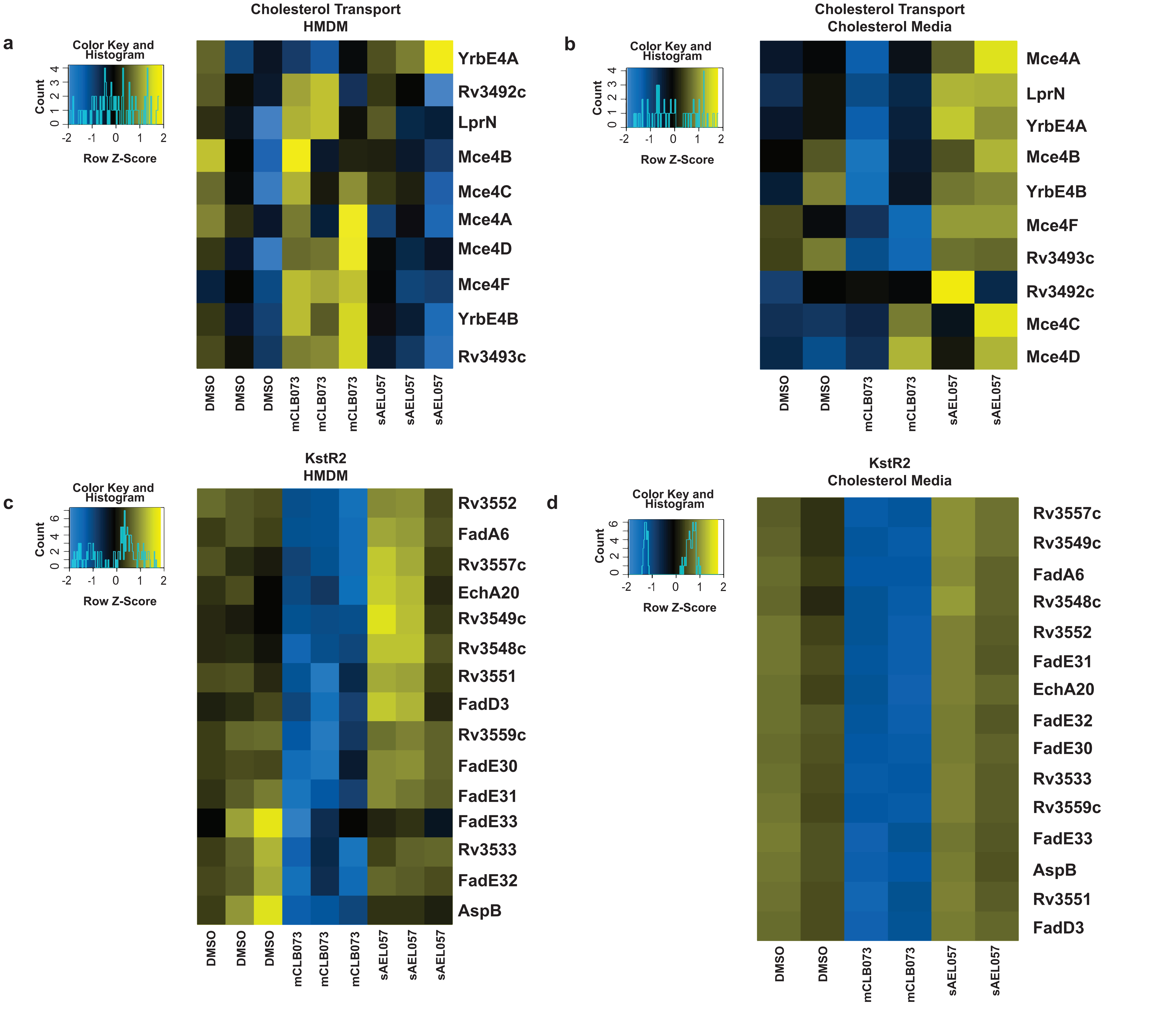

Supplemental Figure 4. sAEL057 treatment doesn't impact expression of cholesterol transport or KstR2 regulon genes in Mtb. Mtb was pre-grown in cholesterol-supplemented media (7H9 + cholesterol) for 2 days prior to addition of sAEL057 at 10x MIC (23 μM). Samples were collected at 4 hours post treatment for RNA extraction. Normalized counts were used for generation of all heatmaps. Heatmap showing relative expression levels for genes related to cholesterol transport for Mtb in HMDMs **a** or cholesterol media **b**. Heatmap showing relative expression levels for genes encoded in the KstR2 regulon for Mtb in HMDMs **c** or cholesterol media **d**.

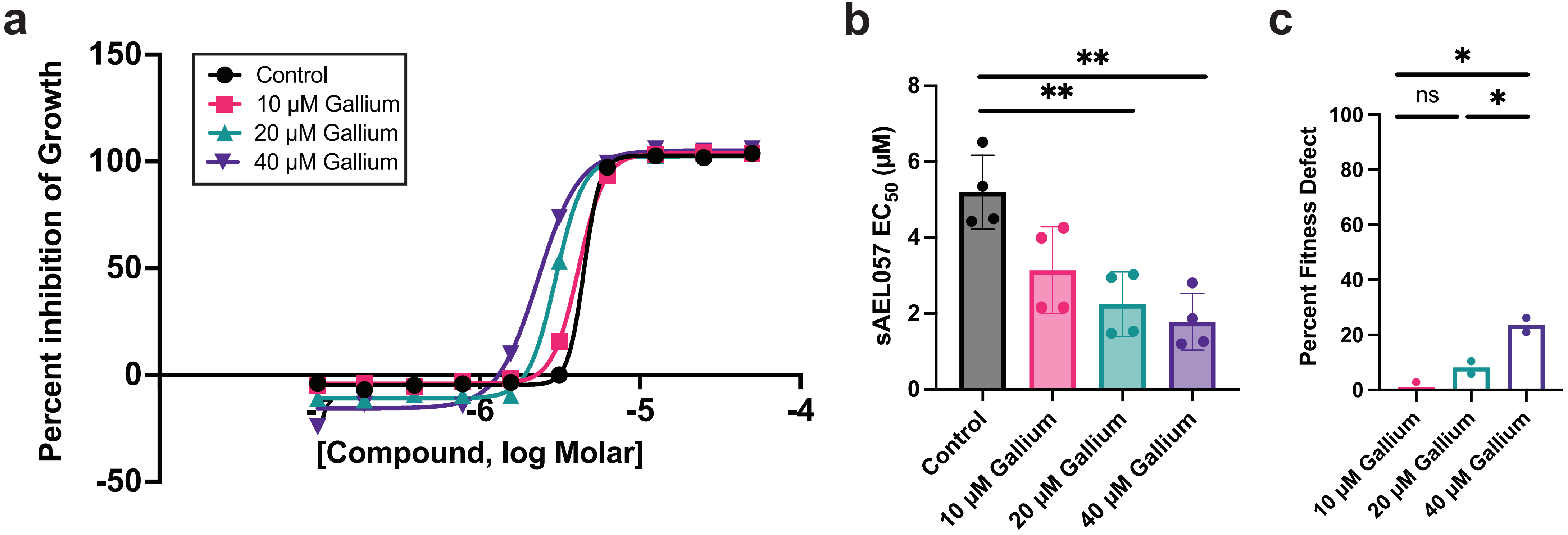

Supplemental Figure 5. Impact of gallium addition on sAEL057 activity in glucose media. Mtb were grown in glucose supplemented media and treated with sAEL057 at 50 μM down to 0.097 μM in dose curve dependent manner. Alamar blue was added at day 9 and results were measured via fluorescence on day 10. **a** Representative graph of percent inhibition curves. Percent inhibition was determined relative to DMSO (0% inhibition) and 20 μM RIF (100% inhibition) controls. n = 2 technical replicates from a representative experiment. **b** EC50 values calculated using non-linear regression analyses (log inhibitor vs response) of percent inhibition curves. n = 4 from 2 replicate experiments. **c** % Fitness defect was calculated by dividing gallium only (without sAEL057) controls by DMSO controls and multiplying by 100 in the given media condition. Statistical significance was assessed using a student's unpaired t-test. Error bars indicate standard deviation.

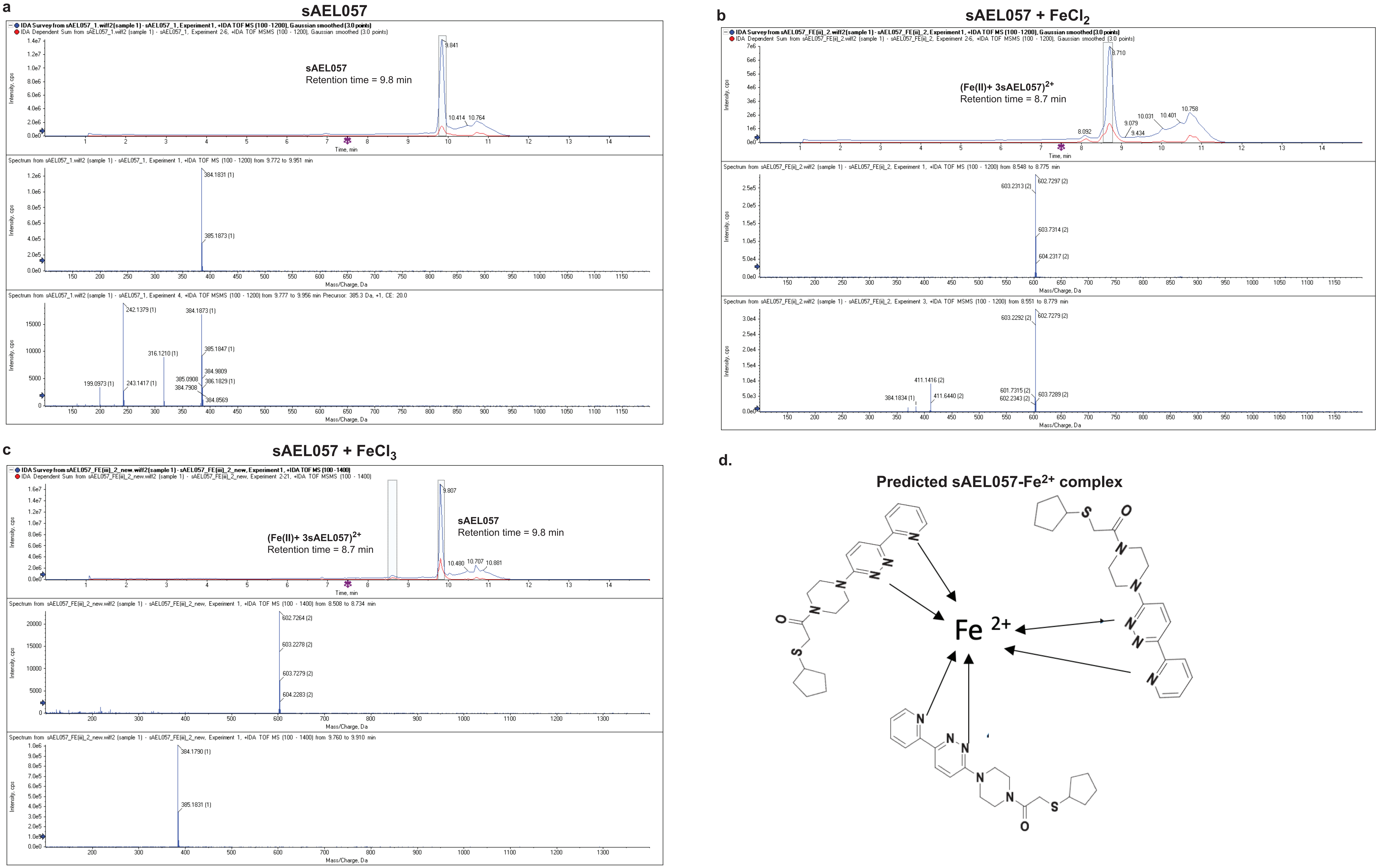

Supplemental Figure 6. sAEL057 binds and forms a complex with ferrous iron. LC-MS/MS spectra of **a** sAEL057 **b** sAEL057 with FeCl2 (ferrous iron source) and **c** sAEL057 with FeCl3 (ferric iron source). **d** Predicted complex based on masses of sAEL057 and Fe2+ which were used to calculate monoisotropic mass.

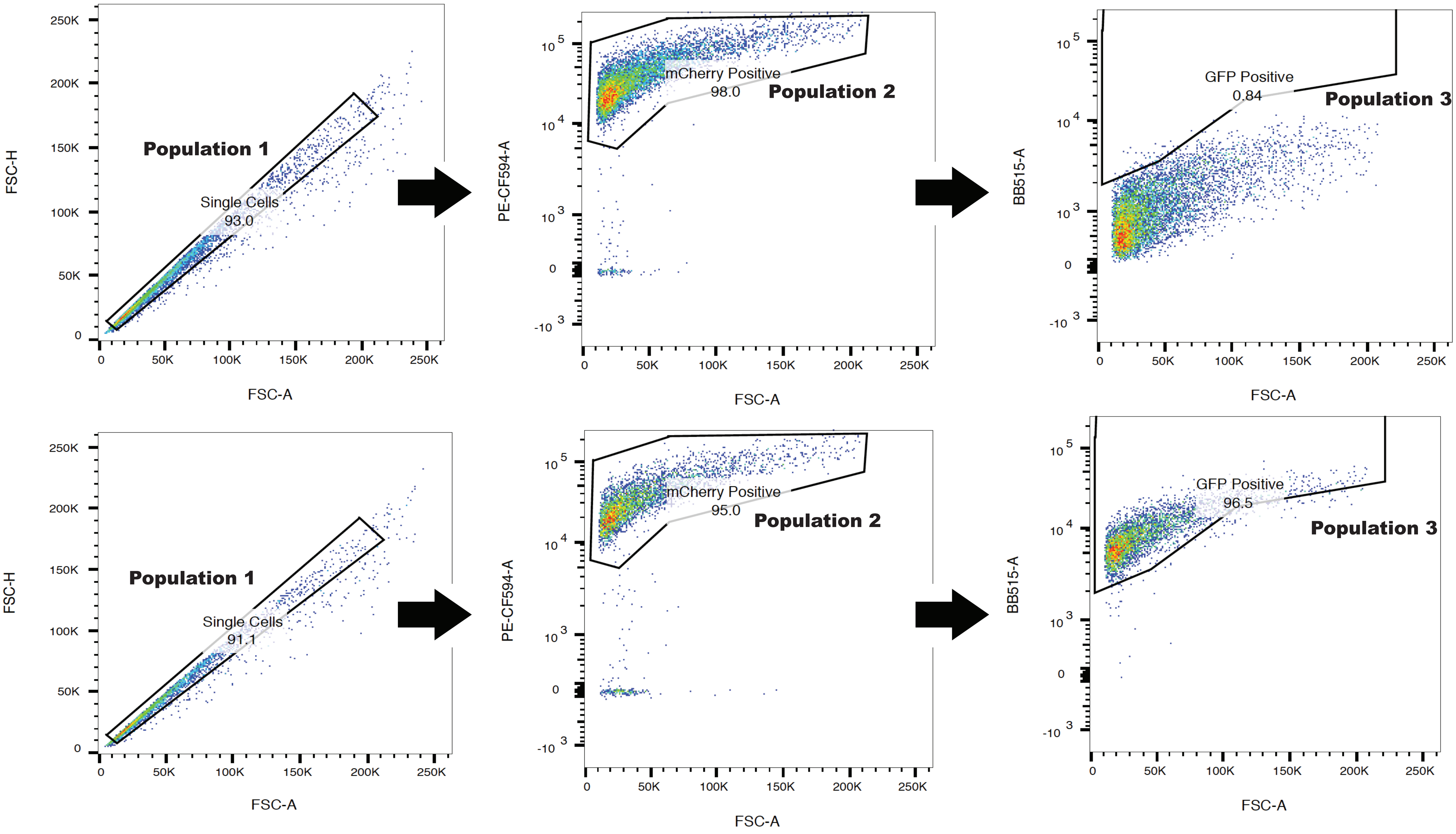

Supplemental Figure 7. Gating strategy for Figure 4. Population 1: Single bacterial cells FSC-H by FSC-A. **Population 2**: mCherry positive bacteria. **Population 3**: GFP positive + mCherry positive bacteria.

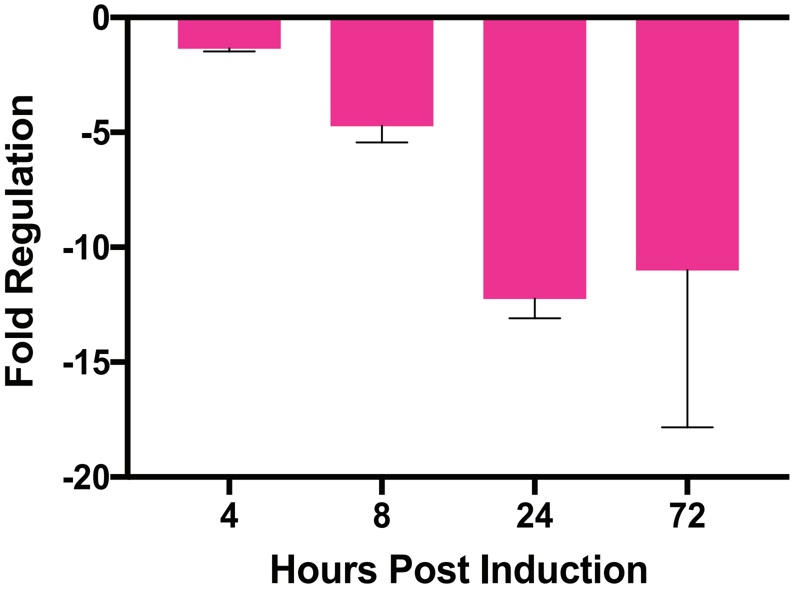

Supplemental Figure 8. Fold regulation of ideR upon addition of ATc. RT-qPCR analyses were performed on an ATc-inducible ideR knockdown Mtb strain. Error bars represent fold change low and fold change high values.

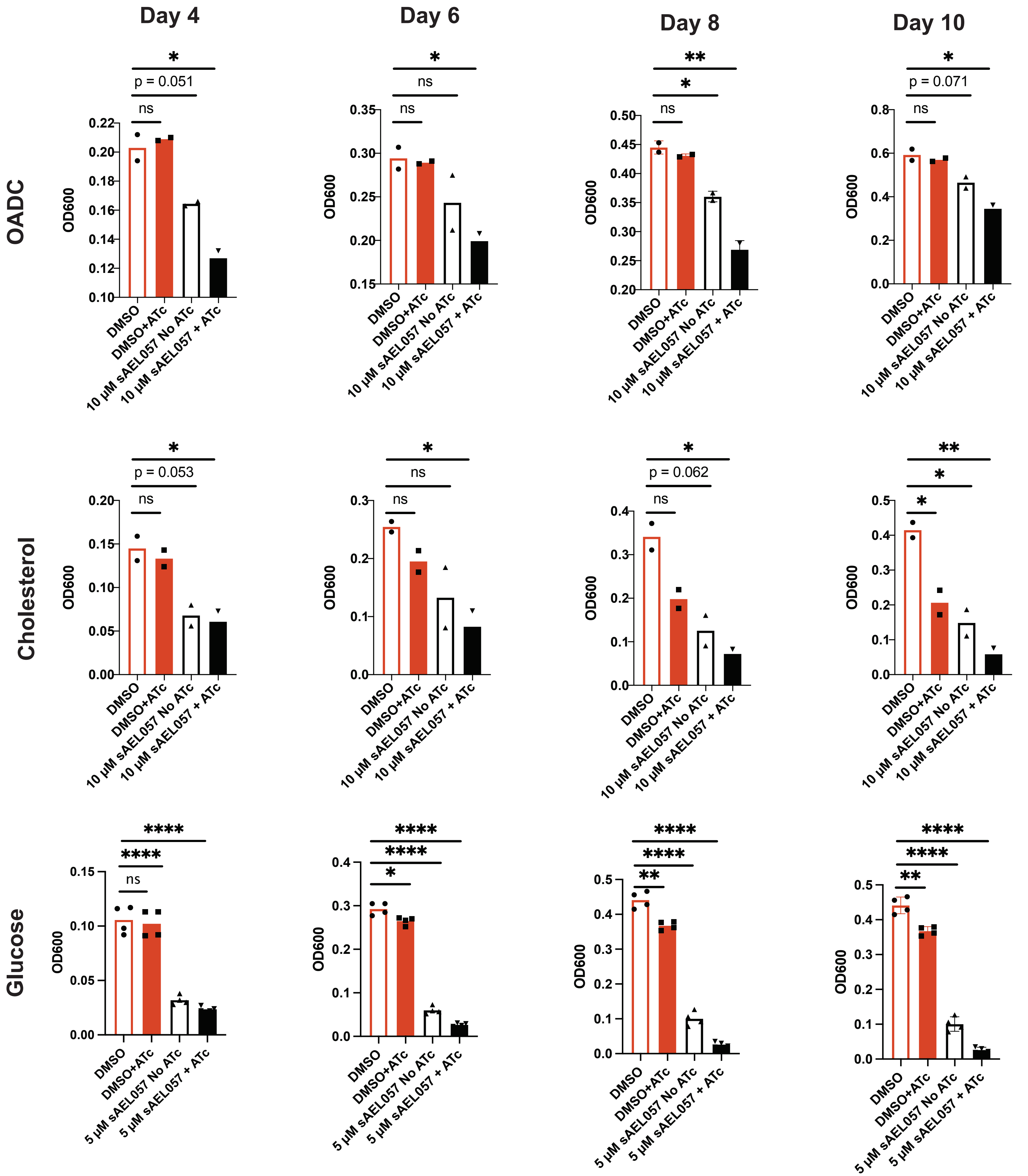

Supplemental Figure 9. Bar plots depicting data from Figure 5 and supplemental Figure 10. IdeR knockdown strain was generated in Erdman Mtb using a CRISPRi construct. The knockdown strain was grown in media supplemented with OADC, cholesterol, or glucose and OD600 values were measured every 2 days. +ATc = induced ideR knockdown, no ATc = uninduced controls. Red bars: DMSO controls (no sAEL057). Black bars: sAEL057 treated at 10 µM in OADC **a**, 5 µM in cholesterol media **b**, or 5 µM in glucose media **c**. Statistical significance was assessed using a student's unpaired t-test.

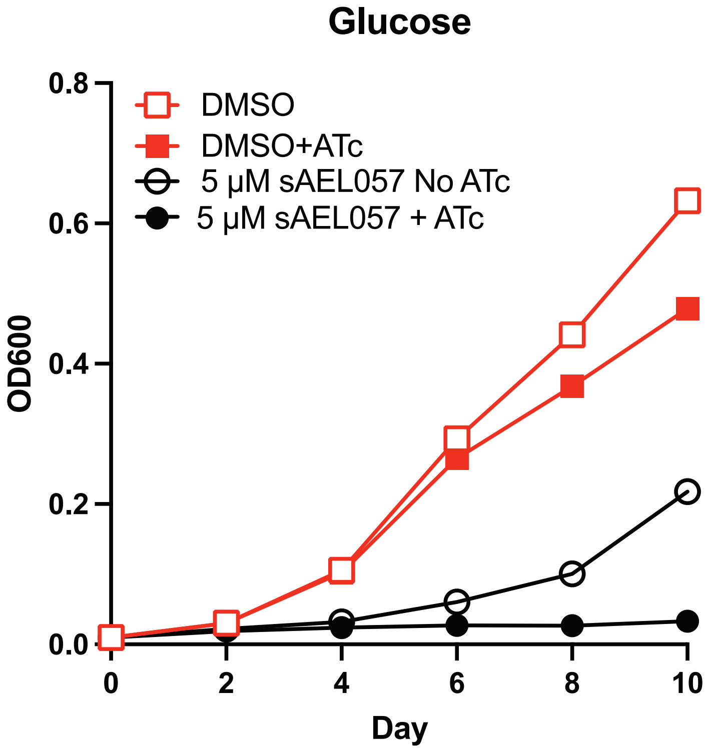

Supplemental Figure 10. Growth phenotype with knockdown of ideR in glucose media. IdeR knockdown strain was generated in Erdman Mtb using a CRISPRi construct. The knockdown strain was grown in media supplemented with glucose and OD600 values were measured every 2 days. +ATc = induced ideR knockdown, no ATc = uninduced controls. Red lines: DMSO controls (no sAEL057). Black lines: sAEL057 treated at 5 μM.

49 Spadina Ave. Suite 200
Toronto ON M5V 2J1 Canada
www.biorender.com

Confirmation of Publication and Licensing Rights

May 1st, 2022
Science Suite Inc.

Subscription:	*Student Plan*
Agreement number:	*WA23V5XITT*
Journal name:	*Communications Biology*

To whom this may concern,

This document is to confirm that Monique Theriault has been granted a license to use the BioRender content, including icons, templates and other original artwork, appearing in the attached completed graphic pursuant to BioRender's Academic License Terms. This license permits BioRender content to be sublicensed for use in journal publications.

All rights and ownership of BioRender content are reserved by BioRender. All completed graphics must be accompanied by the following citation: "Created with BioRender.com".

BioRender content included in the completed graphic is not licensed for any commercial uses beyond publication in a journal. For any commercial use of this figure, users may, if allowed, recreate it in BioRender under an Industry BioRender Plan.

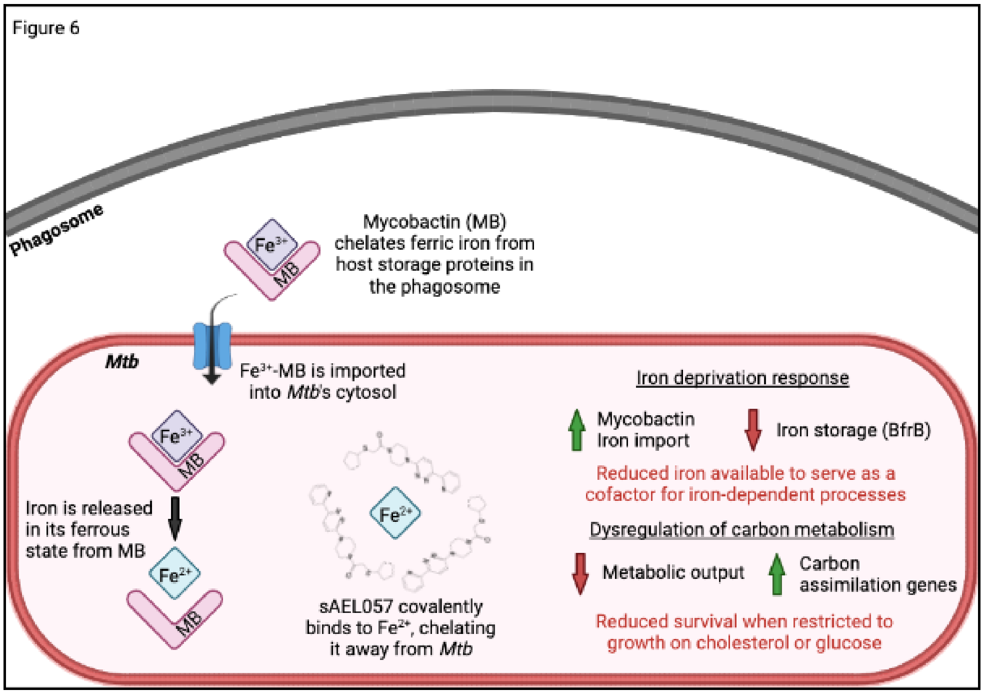

For any questions regarding this document, or other questions about publishing with BioRender refer to our BioRender Publication Guide, or contact BioRender Support at support@biorender.com.
