# Supplementary material for: Iron limitation in M. tuberculosis has broad impact on central carbon metabolism
Source: Commun Biol. 2022 Jul 9;5:685. doi: 10.1038/s42003-022-03650-z (PMC9271047; doi:10.1038/s42003-022-03650-z)
Supplement: Supplementary file 3 — Description of Additional Supplementary Files [file 42003_2022_3650_MOESM3_ESM.pdf]

Description of Additional Supplementary Files

File name: Supplementary Data 1
Description: Source data/p-values for main figures.
